# An ensemble learning model for continuous cognition assessment based on resting-state EEG

**DOI:** 10.1038/s41514-023-00129-x

**Published:** 2024-01-02

**Authors:** Jingnan Sun, Yike Sun, Anruo Shen, Yunxia Li, Xiaorong Gao, Bai Lu

**Affiliations:** 1 Department of Biomedical Engineering, Tsinghua University, 100084 Beijing, China; 2 Department of Biomedical Engineering, Johns Hopkins University, Baltimore, MD 21218 USA; 3 Department of Neurology, Tongji Hospital, School of Medicine, Tongji University, 200092 Shanghai, China; 4 School of Pharmaceutical Sciences, IDG/McGovern Institute for Brain Research, Tsinghua-Peking Joint Center for Life Sciences, Tsinghua University, 100084 Beijing, China; 5 Beijing Academy of Artificial Intelligence, 100080 Beijing, China

**Keywords:** Diagnosis, Cognitive ageing

## Abstract

One critical manifestation of neurological deterioration is the sign of cognitive decline. Causes of cognitive decline include but are not limited to: aging, cerebrovascular disease, Alzheimer’s disease, and trauma. Currently, the primary tool used to examine cognitive decline is scale. However, scale examination has drawbacks such as its clinician subjectivity and inconsistent results. This study attempted to use resting-state EEG to construct a cognitive assessment model that is capable of providing a more scientific and robust evaluation on cognition levels. In this study, 75 healthy subjects, 99 patients with Mild Cognitive Impairment (MCI), and 78 patients with dementia were involved. Their resting-state EEG signals were collected twice, and the recording devices varied. By matching these EEG and traditional scale results, the proposed cognition assessment model was trained based on Adaptive Boosting (AdaBoost) and Support Vector Machines (SVM) methods, mapping subjects’ cognitive levels to a 0–100 test score with a mean error of 4.82 (<5%). This study is the first to establish a continuous evaluation model of cognitive decline on a large sample dataset. Its cross-device usability also suggests universality and robustness of this EEG model, offering a more reliable and affordable way to assess cognitive decline for clinical diagnosis and treatment as well. Furthermore, the interpretability of features involved may further contribute to the early diagnosis and superior treatment evaluation of Alzheimer’s disease.

## Introduction

Cognitive decline is an outward manifestation of neurological decline caused by factors other than natural aging^1^. The causes of cognitive decline include but are not limited to, many neurological disorders such as Alzheimer’s disease, epilepsy, cerebrovascular disease, tumors, other injuries or illnesses such as trauma, diabetes, HIV, thyroid abnormalities, etc.^2^. According to WHO estimates, people suffering from cognitive decline will reach 152 million worldwide by 2050.

The main symptoms of cognitive decline are 1. memory loss. 2. repetitive questioning. 3. abstract logic impairment. 4. language impairment. 5. loss of hobbies and interests. 6. emotional deficits and moodiness. 7. reduced judgment. 8. spatial orientation impairment. 9. misplaced objects. 10. comprehension impairment^3–6^. Cognitive decline places a considerable economic burden on society due to its overall impairment of a person’s ability to function autonomously. Early detection of cognitive decline has become the key to its treatment as many studies have shown that early intervention is relatively effective.

The current diagnosis of cognitive decline relies heavily on scales^7,8^. Alzheimer’s disease, a primary neurodegenerative disease, is one of the leading causes of cognitive decline and even dementia^9^. However, although the academic community often considers scale tests as the ‘gold standard’ for clinical assessments, scale tests themselves indeed have many shortcomings^10^. First, scale tests might not capture the full spectrum of cognitive impairments, especially subtle changes that could be crucial for early diagnosis^11^. Second, they can be influenced by a patient’s educational level, cultural background, or even their emotional state during the assessment^12,13^. Sometimes the results might even be deliberately manipulated. Moreover, repeatability can become an issue; patients might perform better during subsequent tests merely due to familiarity with the test format rather than genuine cognitive improvement^14^. In addition, subjective interpretation by the examiner can also introduce variability in the results. With these inherent limitations, it becomes evident that a more objective and comprehensive method, such as PET and EEG-based assessments, could provide a more accurate picture of a patient’s cognitive health.

EEG has many applications in cognitive decline research as a high temporal resolution neural signal that can be easily acquired^15–17^. Various temporal, spatial, and frequency-based EEG computational features are currently available to evaluate cognitive decline at different levels^18,19^. EEG, as an objective manifestation of the neurological state, will significantly reduce the potentially in cognitive assessments. Moreover, mathematical models based on EEG features will enable a continuous mapping of the macro cognitive state, thereby achieving a more refined evaluation compared to scales^20,21^.

**Fig. 1:**
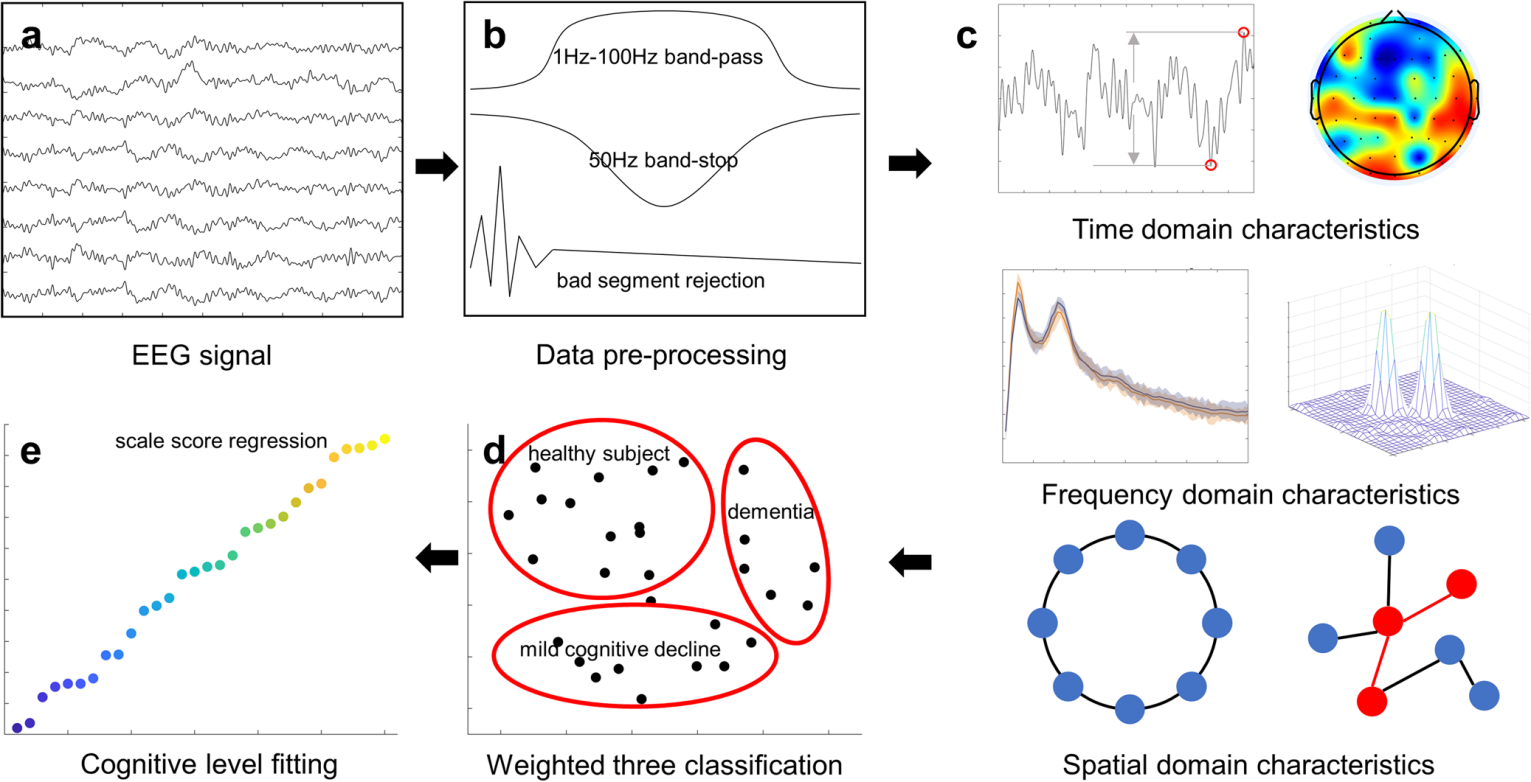
Constructing a continuous cognitive evaluation model. **a** Raw signal EEG signal input. **b** Signal pre-processing, including ring segment rejection, removal of high-frequency noise, low-frequency drift, and industrial frequency interference. **c** Extraction of valid information in the signal from three perspectives: time, frequency, and space domain. **d** Feature filtering to reduce redundant information, triple classification of HC, MCI, and dementia using SVM + AdaBoost method. **e** Cognitive evaluation of the model using cognitive scale test data for regression constraint construct continuum.

While there exists a robust body of research on cognitive segmentation^22,23^, the pronounced noise and substantial variability in resting-state EEG signals make continuous cognitive assessment a challenging endeavor. Nowadays large models are currently a promising method for information modeling, data volume poses a significant challenge in the field of EEG. Before building a vast and reliable dataset, feature engineering remains an effective and feasible approach. Considering that numerous previous studies have explored the differences between patients with cognitive decline and normal subjects from various perspectives of EEG^24–26^, such as time domain, spatial domain, and frequency domain, and have provided different interpretations. The results indicate that time, space, and frequency can each extract distinct information embedded in the EEG. Hence, combining this information seems likely to yield better cognitive assessment results. To extract sufficient information from the complex resting-state EEG, researchers inevitably need to combine as many features as possible. Therefore, this study, using scale scores as the standard, employs the Adaboost method to integrate as many features as possible to achieve continuous cognitive assessment. Furthermore, introducing a plethora of features allows the research to investigate the contributions of distinct features to the modeling outcomes, thereby offering a more comprehensive understanding of the patterns in cognitive decline (Fig. 1).

## Results

### Cognitive scale results

**Fig. 2:**
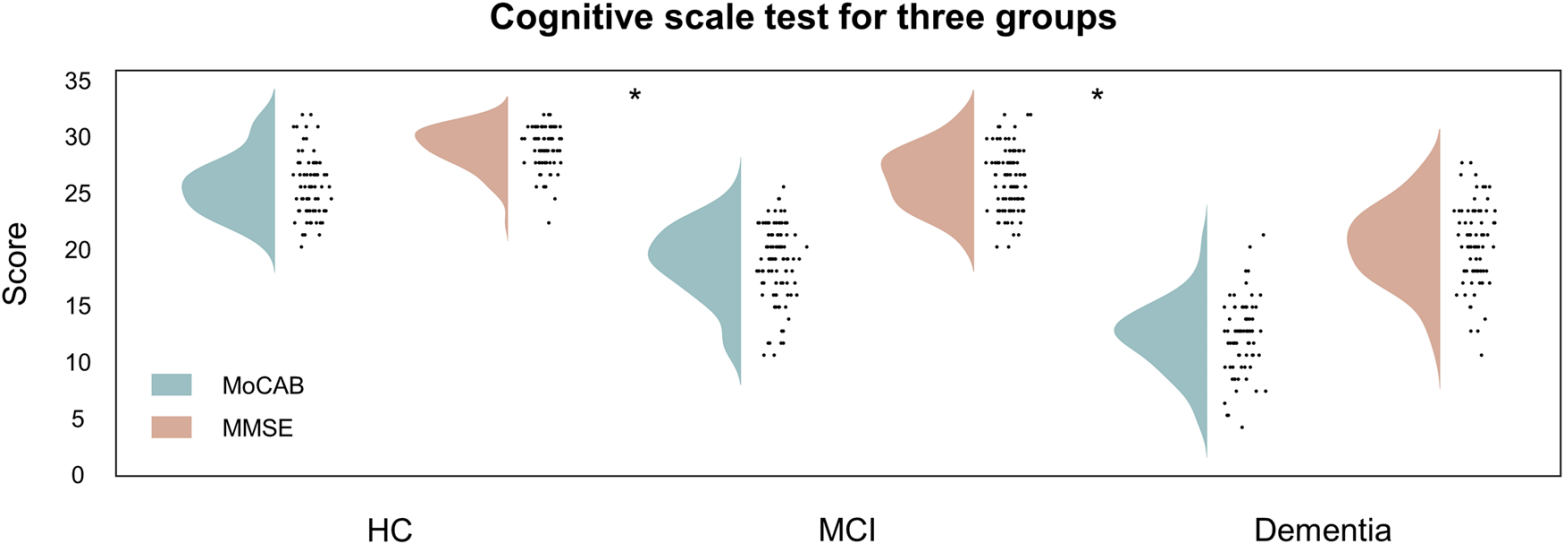
Cognitive scale test for three groups. The box plot shows that the two cognitive scale outcome scores gradually decrease with decreasing clinical cognition, and there is a significant difference between them (MoCAB: HC and MCI, *p* = 9.22*e^−25^; MCI and Dementia, *p* = 1.63e^−21^; MMSE: HC and MCI, *p* = 6.42*e^−11^; MCI and Dementia, *p* = 7.58*e^−21^). (The statistical test was a Mann–Whitney U Test with *p* < 0.05 implying a significant difference).

The study analyzed the cognitive scale test results for the three subject groups (Table 1 and Fig. 2) and discovered that the two-scale tests indicated a decreasing trend in scores with decreasing cognitive capabilities. The mean MoCAB scores for the HC, MCI, and Dementia groups were 24.39, 18.01, and 10.68, respectively, while the MMSE scores were 27.47, 25.18, and 17.86, respectively. It can be seen that as the level of cognition decreases, the range of distribution of the scale test results increases. This suggests that even among those with a clinical diagnosis of dementia, a few subjects have high-scale test scores, which may be associated with high levels of schooling, difficult work, etc. As a result, depending exclusively on scale results carries some risk.

**Table 1:** Classified expressions of different characteristics.

Weak classifier	Single classifier accuracy (baseline)	Final weights	The final number of features
Fractal characteristics	0.44 (0.33)	0.02624	34
Complexity measures	0.56 (0.33)	0.14783	26
Microstate feature	0.67 (0.33)	0.31355	125
Spectral characteristics	0.51 (0.33)	0.11679	113
Bispectral features	0.52 (0.33)	0.10355	82
Network Properties	0.64 (0.33)	0.22268	95
Phase synchronization	0.47 (0.33)	0.07846	37

### EEG patterns of cognitive decline

**Fig. 3:**
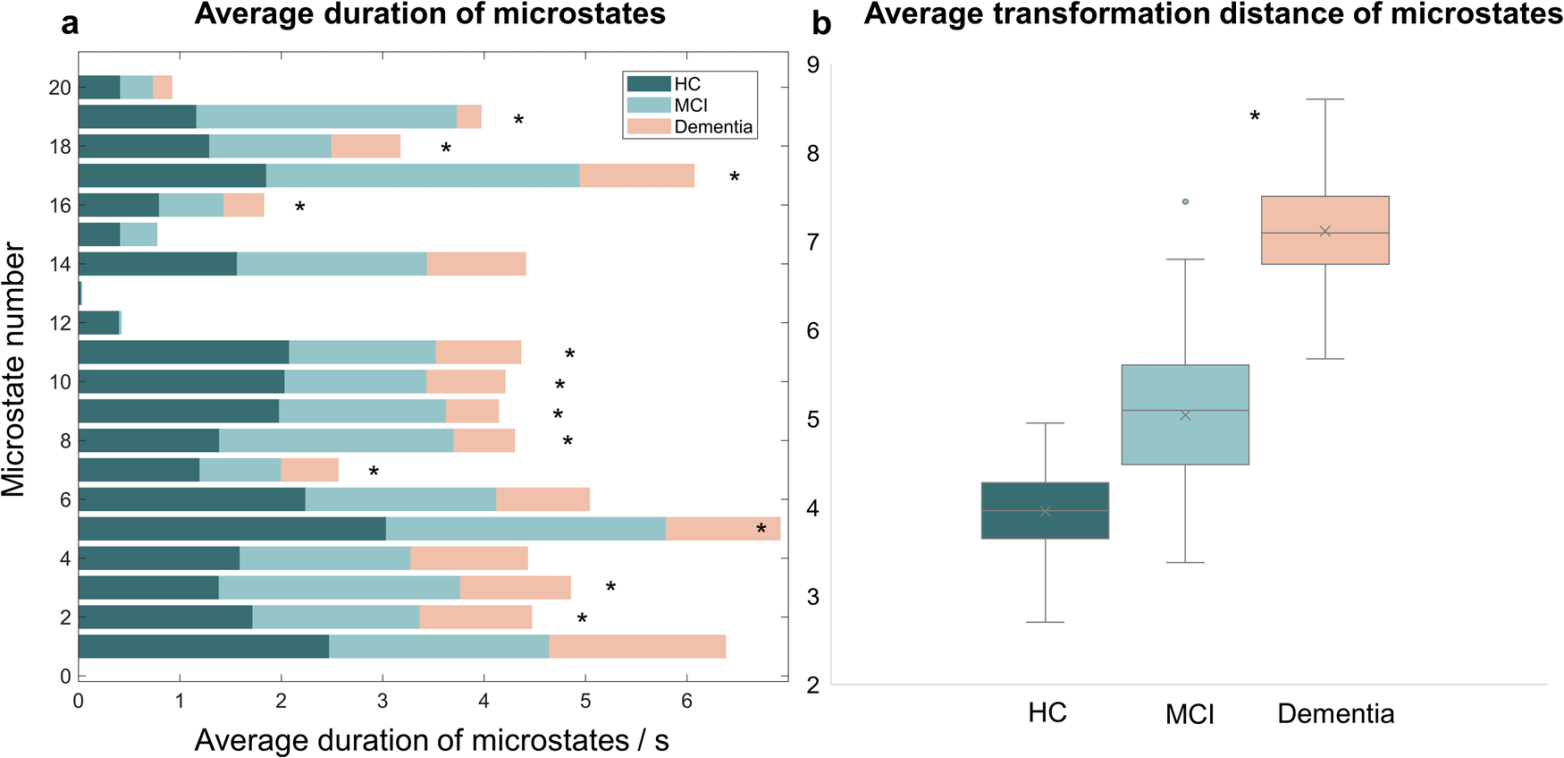
General chronological characteristics of microstates in the three groups. The mean duration and mean state transition distance were calculated for each microstate. **a** It was found that cognitive decline caused the mean duration of states to be shorter, with results showing that HC and dementia all showed significant differences in mean duration for states 2,3,5,7,8,9,10,11,16,18,19 (*p* < 0.05, using ANOVA analysis followed by Bonferroni correction). **b** The mean transition distance showed that cognitive decline might cause an increase in state transition distance. This result was 3.97 for HC, 5.26 for MCI, and 7.06 for dementia, with a significant difference between MCI and dementia (*p* = 0.032).

In the time dimension, cognitive impairment appears to have destabilized network activity (Fig. 3). This implies shorter state durations and more significant gaps between states. In the resting state, the average brain is in a dynamic and stable process that involves constant switching between many modes. The findings, on the other hand, suggest that dementia patients are missing states 12 and 15 (Supplementary Fig. 1), which could be related to structural damage produced by brain atrophy and neuronal death. From a translational standpoint, cognitive deterioration decreased state duration, implying more translations and systemic dissociation. When the average transformation distance results are combined, it is known that brains with cognitive impairment appear to be more prone to state leaps rather than continuous and smooth evolution. The temporal representation results hint at cognitive deterioration, rendering the conscious experience discrete, which may also be associated with impaired central control and a lack of attention.

**Fig. 4:**
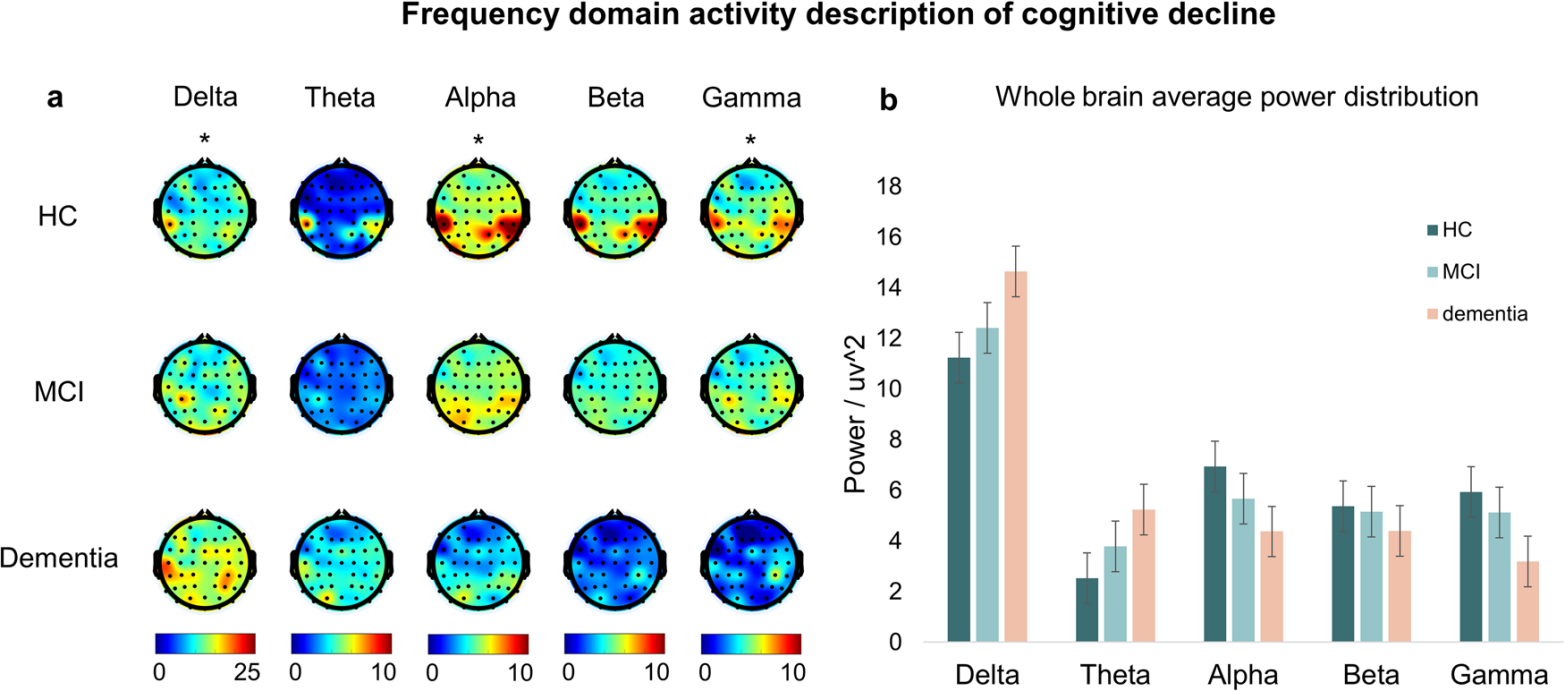
Frequency domain description of cognitive decline. The frequency characteristics and power distribution patterns of the three groups of people with cognitive decline were analyzed according to band power. **a** The power in the delta and theta bands gradually increased with cognitive decline, while the power in the alpha, beta, and gamma bands gradually increased. **b** The delta band MCI was significantly different from Dementia (MCI: 12.42 uv^2^, Dementia: 14.65 uv^2^ and *p* = 0.014), the alpha band HC being significantly different from MCI (HC: 6.94 uv^2^, MCI: 5.66 uv^2^ and *p* = 0.032) and MCI from Dementia (MCI: 5.66 uv^2^, Dementia: 4.37 uv^2^ and *p* = 0.027), and the gamma band MCI being significantly different from Dementia (MCI: 5.13 uv^2^, Dementia: 3.18 uv^2^ and *p* = 0.012). (The statistical test was a two-sample t-test with *p* < 0.05 implying a significant difference).

Regarding the frequency domain dimension, cognitive decline implies an overall slowing of activity. A decrease in high-frequency activity and an increase in low-frequency activity are typical of the frequency domain (Fig. 4). The change from the HC to MCI stage is relatively moderate. In contrast, the change from MCI to dementia is more pronounced. In terms of the spatial distribution of power, the temporoparietal lobe of HC has relatively strong activity in the resting state, while MCI and dementia have weaker activity. Overall activity in HC is highly homogeneous, while frontal lobe activity is significantly reduced in dementia.

**Fig. 5:**
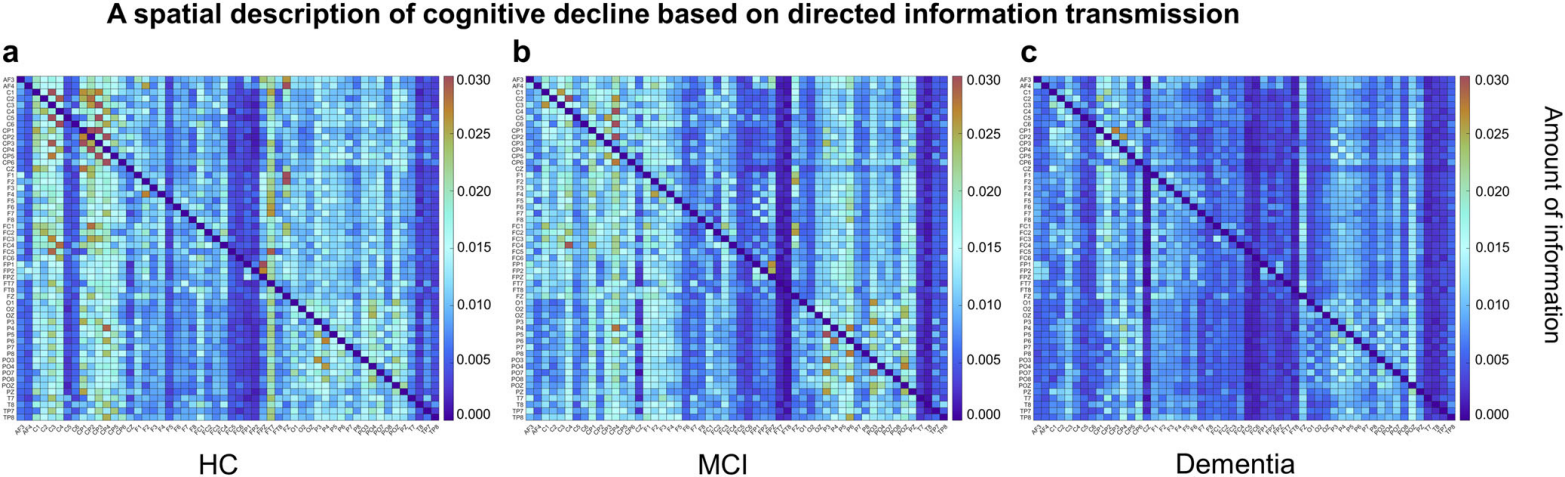
Directed transform function for three groups of people. DTF-based directed information flow, where each sub-square represents the flow of information from the column labels to the row labels. It can be seen that overall information transfer diminishes as cognition declines, **a** with a mean information strength of 0.0099 for HC, **b** 0.0074 for MCI, and **c** 0.0032 for dementia, with a significant difference between MCI and Dementia (*p* = 0.026). (The statistical test was a two-sample t-test with *p* < 0.05 implying a significant difference).

Observed in the spatial dimension, the decrease in overall information transfer brought about by the cognitive decline implies a silent and inactive network (Fig. 5). The results point to a higher outflow of information from the central regions of the network (C1, C2, C3, C4, CP1, CP2) in the resting state, which may imply central control and moderation. This phenomenon diminishes with decreasing cognition, and the average information transfer across the network decreases. In addition, it was observed that cognitive decline did not imply a monotonic network degeneration and that enhanced information between specific regions was observed from the HC to the MCI stage, e.g., a slight increase in outflow information in C5, C6, FP1, FP2 compared to the HC network (4.93% increase on average).

**Fig. 6:**
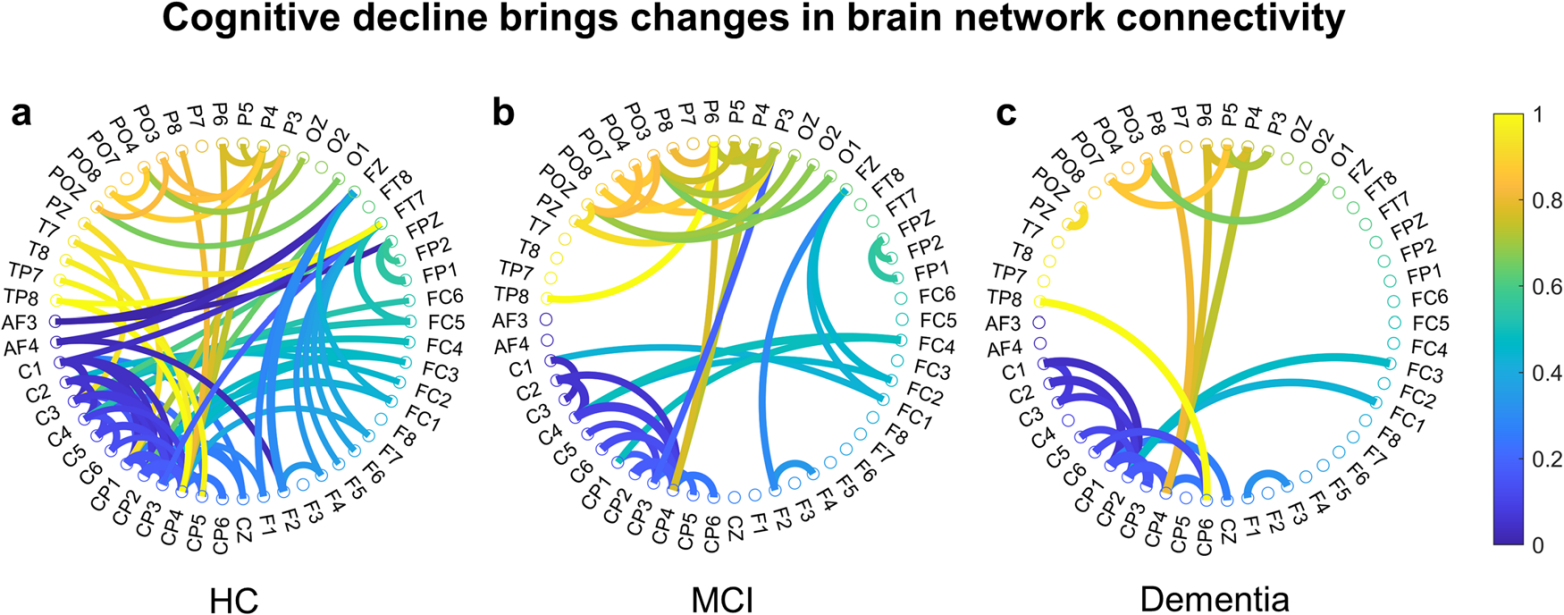
Schematic diagram of the major connections for the three groups. As can be seen from the connection, the number of major network connections decreases as cognition moves downwards. **a** The HC network containing 82 connections, **b** MCI network containing 43 connections, **c** the dementia network only 25 connections. There were significant differences between HC and MCI (*p* = 0.043) and MCI and dementia (*p* = 0.041). The results indicate a tendency for solid connections to remain and weak connections to decrease or disappear during cognitive decline. In the figure, the color indicates the normalized connection strength. (The statistical test was a two-sample t-test with *p* < 0.05 implying a significant difference).

Observed from the perspective of spatial networks, cognitive decline reduces network connectivity. This section calculates the sum of incoming and outgoing information between any two nodes as the strength of the connection between these two nodes. The research filters the network’s major connections according to a criterion of greater than 80%. It presents them normalized (Fig. 6). The results indicate that the number of major connections in the network decreases with cognitive decline and follows the principle of retaining strong connections and reducing weak ones.

### Cognitive decline prediction

In this work, many features were extracted from the EEG of subjects with different cognitive levels in three dimensions: time domain, space domain, and frequency domain. Features are composed of eight weak classifiers according to the feature types, and the classification effect of each feature individually is shown in Table 1. The results point to a relatively good classification performance for the microstate phase pipe features and a classification accuracy of only 0.64. This indicates that the amount of information carried by a single-dimensional feature is insufficient for the triple classification task of HC, MCI, and dementia.

**Fig. 7:**
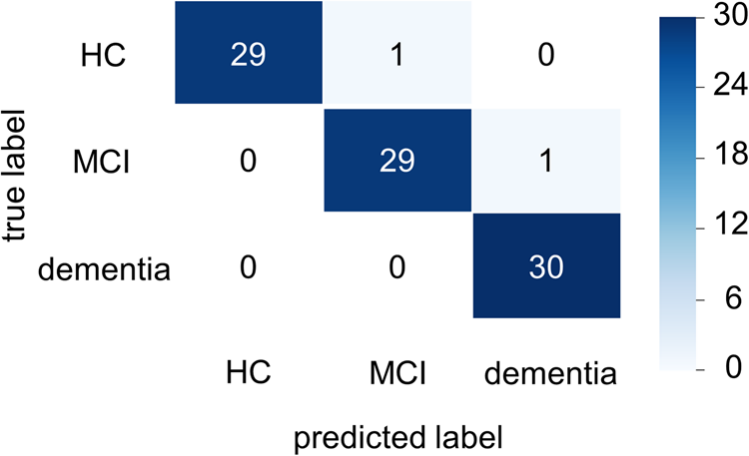
Confusion matrix with three classifications. The confusion matrix shows an accuracy of 0.93 for the triple classification, with two misclassified samples out of 30 tested. No HC was misclassified as demented, or demented was misclassified as HC.

The strong classifier consisting of 7 weak classifiers after feature filtering and a combination of 7 weak classifiers could achieve a better triple classification, and a classification accuracy of 0.9333 (28 out of 30) was obtained on the test set. As can be seen from the confusion matrix (Fig. 7), all patients with dementia were correctly predicted. One HC was predicted as MCI, and one MCI was predicted as dementia. While studies usually consider HC, MCI, and dementia as a continuum of development, this study also looked further at the cognitive test scores (mean MoCAB and MMSE) of the mis-scored sample. The cognitive test score for the misclassified HC sample was 23, while the MCI misclassified sample had a cognitive test score of 18. Considering that the full score on the cognitive test is 30, the lowest score for the HC sample was 22, while the lowest score for the MCI sample was 15, both misclassified samples were also at the lower end of their respective categories.

**Fig. 8:**
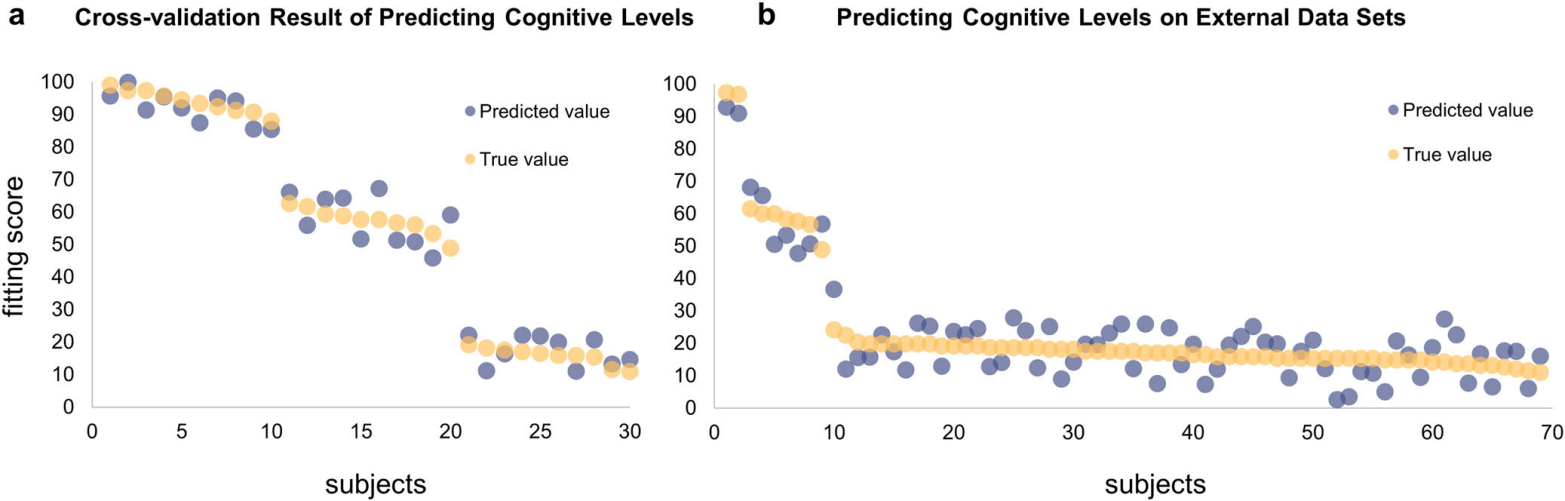
Final cognitive level prediction results and Validation. The integrated learning approach combining resting-state EEG and cognitive scales has an excellent predictive effect on the cognitive level, **a** with results indicating a mean prediction error of 4.82 (error rate less than 5%) on a scale of 1–100 (with 100 representing the healthiest and 1 representing severe dementia). The model also predicted subjects at different levels with uniform errors (left figure). **b** The validation results of the predictive model on an external dataset yielded an error of 6.12 (right figure).

A continuous cognitive evaluation model can be obtained by restricting and weighing the outcomes of the generated trichotomous model based on the results of the cognitive scale tests (MoCAB and MMSE). After mapping the true and predicted scores to a score between 1 and 100 using the procedure section of the algorithm, when making final predictions for the cognitive levels of 30 individuals through cross-validation, the average error obtained was 4.63 (Fig. 8). Examining the two misclassified samples again, the true cognitive score for the sample classified as MCI was 62.70 and the predicted value was 66.11, while the true value for the sample misclassified as dementia was 33.15 and the anticipated value was 35.54. The results demonstrate that after the continuous evaluation, the cost of misclassification was lowered, and the prediction of the neighboring category shows that there may be a cognitive effect—the forecasts for the neighboring categories point to a potential pattern of perceived deterioration in this sample. Overall, the findings indicate that the approach accurately predicts cognitive function from resting EEG. The mean prediction error was 4.82 on a scale of 1–100 (100 being the healthiest and one being seriously demented), resulting in a less than 5% error rate. To assess the generalization performance of the model, the study also conducted tests on an external dataset, yielding an average error of 6.12 (Fig. 8).

## Discussion

This study proposes a method that can predict the cognitive level of subjects based on resting-state EEG and validates the feasibility of the method on EEG dataset of people with different cognitive levels, obtaining predictions error of 4.82 (<5%). The evaluation model has easy input data collection advantages, low algorithm complexity, and high model interpretability. The method may contribute to the early screening of cognitive decline, the assessment of the effectiveness of therapeutic interventions for neurodegenerative diseases associated with cognitive decline, mainly Alzheimer’s disease, the development of drugs for related diseases, and the evaluation of drug efficacy. In the context of current cognitive assessment methods, scales exhibit limited sensitivity to subtle changes and suffer from ceiling effects, while neuroimaging tools are constrained by environmental factors and expensive contrast agents, and the collection of biological markers is challenging. Based on the findings of this study, electroencephalography (EEG) may potentially offer contributions in this direction.

One question worth discussing is why many single types of features do not distinguish the three groups well simultaneously, while once they are combined in a specific form, satisfactory classification accuracy can be obtained. Schapire proves that if the target is weakly learnable, it must be possible to make it strongly learnable in some form based on the significant number theorem^27^. In practice, to achieve this goal, the errors of each weak classifier need to be independent of each other. That is, different weak classifiers can provide different information to achieve the error reduction of strong classifiers. The core purpose of this study’s sample weight adjustment strategy using the Adaboost method is to ensure an even distribution of correctly classified samples, ultimately making the number of correctly classified samples more than the number of incorrectly classified samples. The results of the weak classifier for the predicted samples point out that the starting classification distribution of the samples in this problem is relatively uniform, making it easier to improve the accuracy during further iterations of the weights (Supplementary Fig. 2). In addition, the study also correlated the classification result labels of each weak classifier (Supplementary Fig. 3). The results showed that each weak classifier did not perform the same, and there was no weak class of classifiers with similar information contributions that could be replaced or removed.

Specifically, in this study, the reason for the success of integrated learning is that the different features pinpoint different levels of variation and increase the amount of valid information at each iteration. The results show that the microstate sequence-related features perform relatively well in the differentiation of the three populations, which may be due to the high temporal resolution of the EEG that allows for the mining of fine-grained systemic transformations that are highly correlated with disease and abnormal activity^28^. Spatial network features performed best in distinguishing MCI from dementia but were average for distinguishing HC from MCI. This may be because the structural changes accompanying early neurological decline are not very significant, while the structural differences become increasingly significant as the system collapses^29^.

Another advantage of this work is that the data used contains three different acquisition devices (NeuroScan, Neuracle, BP). The combination and processing based on the feature level are not device-dependent and do not require device consistency (Supplementary Table 1 and Supplementary Fig. 4), so a multi-device acquisition dataset has no impact on the model building of this work. This study also researches the distribution of the true and predicted values of the prediction sample by an amplifier to ensure the approach is pervasive and cross-device capable. The results showed that the distribution tests between the true and predicted values of the test samples from the three amplifiers did not show any difference (*p* > 0.1). Furthermore, the absolute prediction error for all three amplifiers is close to the absolute prediction error for the full sample (Total error: 4.63; NeuroScan error: 4.13; Neuracle error: 4.33; BP error: 5.23). The advantage of multiple devices, however, is the increased diversity of data, which also contributes to the generalizability and credibility of the model and establishes a sound basis for future algorithm applications^30^.

Considering the strong correlation between cognitive decline and aging, the contribution of age in the model obtained by fitting based on cognitive decline is also worth discussing; after all, the study does not expect to obtain only one age predictor. For this reason, this study used the same Adaboost method to fit the age and predict the test sample’s age. After obtaining the predicted age corresponding to the actual clinical labels of the subjects, it was found that the results of the model fitting seemed to have some age correlation. However, it can be seen from the figure that the results of the model fit do not all depend on age, as there are normal subjects with a high predicted age and demented subjects with low predicted age (Supplementary Fig. 5).

The method has strong interpretability, which is crucial for clinical purposes. The results of the weak classifier show that the time domain features are relatively well classified, followed by the space domain features and again by the frequency domain features. This fully reflects the high temporal resolution of the EEG signal, which also indicates that the three categories of HC, MCI, and dementia, that is, the process of cognitive decline, differ more significantly in the transformation of neural activity patterns in the temporal dimension. This part of the results is also reflected in the paper. In particular, early cognitive decline, which is poorly identified subjectively, can be better detected by this model.

This study also describes the pattern of cognitive decline in three dimensions, spatiotemporal and frequency, to enhance the interpretability of the evaluation system. Combining the results of this study with previous work, the study concluded that cognitive decline follows four rules: the stronger ones always prevail., non-monotonic changes, decreased efficiency, and reduced stability.

One of the key principles elucidated by this study is the concept that ‘the stronger ones always prevail.’ This principle underscores the non-uniform nature of neural system changes during cognitive decline. It suggests that certain strong connections and active neural regions endure the degeneration process more effectively. These robust connections and active areas may represent an individual’s essential cognitive capacity. For example, preserving parietal connections^31^ may be related to central control and basic perception.

It is important to recognize that cognitive decline does not follow a monotonic trajectory. Instead, it exhibits dynamic characteristics, including the potential for neural repair and compensation, especially in the early stages^32^. As demonstrated by our results, cognitive decline accelerates over time, with the transition from Mild Cognitive Impairment (MCI) to dementia displaying a notably faster decline rate compared to the transition from Healthy Controls (HC) to MCI. Intriguingly, there are instances of additional connectivity and enhanced information observed during the HC to MCI transition. This phenomenon may contribute to the limited success of linear predictive models in some previous studies^33^, highlighting the complexity of cognitive decline dynamics.

Decreased efficiency is also a primary phenomenon. Cognitive decline characterizes the degradation of the nervous system, with reduced activity and information implying reduced processing of external input^34^. Some studies on cognitive states have also demonstrated that dementia may lead to increased reaction times, attention deficits, and reduced memory capacity^35^. These phenomena are closely associated with a decrease in the information content of signals and a reduction in complexity, both of which signify a weakening of information transmission capacity and contribute to model evaluation. This correlates with the slowness of response, attention deficit, and repetition of questions and answers exhibited by patients with cognitive decline.

Finally, cognitive decline will reduce stability. The normal brain is a stable dynamic equilibrium system, and cognitive decline disrupts this equilibrium^36^. Combined with the findings, the jumps in state and the diminished persistence indicate a system disruption. Some patients with cognitive decline exhibit emotional abnormalities, and communication difficulties may be associated with this.

In reference to previous studies on EEG data^24,37^, it is generally considered that a sample size of several dozen data points carries significant statistical significance. In this study, a total of 252 participants were included, resulting in 504 data instances. While this sample size may not be considered large when compared to large-scale models used in image and text processing, it is sufficient for addressing the research questions and methods employed in this study. Furthermore, the inclusion criteria for patients strictly adhered to clinical labels provided by the hospital, ensuring a relatively reliable assessment of patient progress. However, it is important to acknowledge that there are still some limitations in this study due to data constraints. The application of the AdaBoost method based on feature engineering introduces a relatively high computational complexity, which may lead to practical challenges during real-world implementation. In addition, the inherent nature of the AdaBoost method prioritizes the handling of outliers, potentially affecting the model’s ability to cope with noise.

In fact, this study is primarily based on cross-sectional data to characterize signals and features across different populations for the development of predictive models. However, conducting a longitudinal tracking of a cohort of participants to investigate cognitive changes and EEG variations within a group over time would offer an entirely fresh perspective. Such an approach could aid researchers in gaining a deeper understanding of the patterns of cognitive decline and associated neurophysiological changes. While the inclusion of a longitudinal follow-up component in our study presents logistical challenges, it also holds the promise of yielding richer insights into cognitive trajectories and model performance. Future research endeavors could explore the feasibility and potential benefits of such an approach in advancing our understanding of cognitive assessment using EEG.

The implementation of our EEG-based cognitive assessment model holds significant promise for enhancing patient care. Firstly, it could serve as a valuable tool for early detection and monitoring of cognitive decline in individuals, allowing for timely intervention and personalized treatment strategies. By providing quantitative insights into cognitive function, the model could aid clinicians in making informed decisions about treatment plans and interventions. Furthermore, the model’s ability to capture nuanced patterns of cognitive decline, as highlighted in our study, could contribute to more accurate and sensitive assessments of treatment efficacy. Clinicians could use the model to track changes in cognitive function over time, allowing for the precise evaluation of therapeutic interventions and the adjustment of treatment approaches as needed. In terms of clinical decision-making, the model’s predictive capabilities could aid in risk assessment and prognosis estimation, enabling clinicians to provide patients and their families with more informed expectations about the course of cognitive decline.

In summary, this study also presents a potential approach for constructing a neural activity space using samples and labels, such that the mapping can characterize various neural activity changes. With the help of some techniques to increase the signal-to-noise ratio of EEG^38,39^, such a framework could extract information from resting-state scalp EEG more effectively. The cognitive decline mapping proposed in this study is only one part of a larger neural activity space. The construction of such a mapping system will be helpful for neurological function assessment, disease development evaluation, clinical target research, and drug effect testing.

## Methods

### Participants

The participants were patients with complaints of cognitive decline from the Memory Specialist Clinic of the Department of Neurology, Tongji Hospital, Tongji University. The Ethics Committee of Tongji Hospital, Shanghai, China, approved the study. Healthy elderly controls (HECs) with matching sex, age, and education level were recruited from the local community in Shanghai. Patients with MCI and dementia underwent a standard clinical diagnosis including cranial computed tomography (CT) imaging or Magnetic Resonance imaging (MRI) scan and scale testing^40^. All subjects were informed and signed an informed consent form. Patients were required to meet the following criteria: (1) no brain tumor, epilepsy, neurosyphilis, no new cerebral infarction at the time of consultation, and no other central nervous system diseases (infections, clear history of demyelinating diseases, etc.); (2) no major medical or physical diseases such as hepatic encephalopathy or myocardial infarction; (3) no previous history of severe mental disorders, psychoactive substances, or drug abuse; (4) able to cooperate with the examination, complete the full neuropsychological assessment and sign an informed consent form; (5) no contraindications to cranial MRI or electroencephalography. A total of 252 participants were enrolled in this study, including 75 healthy controls, 99 MCI, and 78 patients with dementia (Table 2). In addition, EEG was acquired twice per participant, so the data involved in the model building was 150 healthy subjects, 198 MCI, and 156 dementia patients.

**Table 2:** Subject information.

	HC	MCI	Dementia
Number	75	99	78
Females/males	38/37	53/46	40/38
Age (years)	67.9 ± 13.5	70.6 ± 14.2	72.7 ± 13.8
*p*-value of t-test for age distribution	0.014 (HC and dementia)	0.095 (HC and MCI)	0.021 (MCI and dementia)
MoCAB	24.39 ± 5.39	24.39 ± 5.39	24.39 ± 5.39
MMSE	27.47 ± 3.47	27.47 ± 3.47	27.47 ± 3.47

### EEG data acquisition and pre-processing

EEG data acquisition is performed using a 64-lead EEG recording system from Brain Products (www.brainproducts.com), NeuroScan (www.compumedicsneuroscan.com), and Neuracle (www.neuracle.tech), with electrode positions using International 10–20 EEG Society standards. The equipment sampling rate is 1000 Hz. The electrode contact resistance was less than 5 KΩ. Resting EEG was recorded with eyes open for at least 5 min before the task EEG was performed on all subjects. EEG signal pre-processing processing included pre-positioning electrodes, removal from 50 Hz industrial frequency interference, 0.5–100 Hz band-pass filtering, rejection of useless channels, threshold method to reject bad segments, ICA to remove EMG artifact components and whole brain average re-referencing. EEG signals were processed in this research by MATLAB 2018b platform (ww2.mathworks.cn).

### Validation dataset

In order to assess the robustness and generalization of the model, the study was tested on an external dataset (available at https://figshare.com/articles/dataset/dataset_zip/5450293/1)^41^. This dataset comprises 2 healthy subjects, 7 mild cognitive impairment (MCI) subjects, and 59 dementia subjects, each accompanied by their Mini-Mental State Examination (MMSE) scores as a cognitive assessment. The dataset consists of 19-channel EEG data. To align with the evaluation of the model, channel expansion was performed on the dataset using nearest-neighbor interpolation.

### Cognitive scale tests

Each participant was tested on the Montreal Cognitive Assessment Basic (MoCAB)^42^ and the Mini-Mental State Examination (MMSE)^43^ scales to determine their cognitive level. MoCAB is a quick cognitive impairment assessment tool, and the MMSE is a technique for detecting cognitive and intellectual deterioration in older persons. The scales test the capability of visuospatial, executive, naming, memory, attention, language, abstraction, delayed recall, orientation through charts, answers for questions and choices. Both scales have been translated into several languages and are widely used around the world.

### Time domain feature extraction (fractal features)

The time domain feature extraction mainly considers the time-varying characteristics of the EEG signal. In this part, except for the microstate calculation, 50 trials 2 s segments are segmented for superimposed averaging. Fractal features characterize the waveform of the EEG, and in this work, the fractal features of nine EEGs were calculated. The specific calculations are shown in Supplementary Table 2, assuming an EEG signal of X.

### Time domain feature extraction (signal complexity)

Complexity is a statistical measure of the amount of information carried by a signal, and Lempel-Ziv signal complexity (Lempel and Ziv, 1976) is used in this study. It evaluates the probability of emerging signal patterns and the feature of neural activity transitions. Assume that the EEG signal *X* = [*x*_1_, *x*_2_, …, *x*_*n*_], *x*_*n*_ denotes the amplitude, while *n* denotes the time.

1 $${S}_{n}=\left\{\begin{array}{lc}0&{x}_{n}\le {T}_{1}\\ &{1{{T}_{1}\, < \,x}_{n}\le {T}_{2}}\\&{\vdots }\\ l&\,{{T}_{l}\, < \,x}_{n}\end{array}\right.;{\rm{C}}=\frac{c{\log }_{l}n}{n}$$

In the formula, the original signal (formula 1) is segmented into a new sequence *S* according to l segments. *T*_l_ denotes the threshold value obtained by dividing the difference between the maximum and minimum values of the EEG signal according to l-segment averaging. And then, the normalized complexity of the *S* sequence is calculated^44^, in which *c* is the complexity of sequence *S*.

### Time domain feature extraction (microstate feature)

In this study, the microstate analysis method is used to extract the transformation information of the EEG. In this paper, the segments are segmented according to 100 ms, and the standard deviation of the signal within each 100 ms is calculated as a single microstate. Each segment of EEG data is divided into a total of 1500 microstates. Each 100 ms (0.1 s) characterizes a microstate, and the research selects 150 s of high-quality segments from the recorded 5 min data and divides them into a sequence of 1500 microstates. Finally, 756,000 microstates from all 504 segments of EEG are clustered to obtain a category label for each microstate (divided into 20 classes). The sequence features of each individual microstate sequence were extracted to obtain the state transition information of each data, and the specific calculation features^45^ and methods are shown in Supplementary Table 3. In the table ${S}_{i}$ represents the microstate I, and ${{S}}_{{in}}$ is one element of microstate vectors ${S}_{i}={[S}_{i1,}{S}_{i2,}\ldots ,{S}_{{in}}]$.

### Frequency domain feature extraction

The frequency domain is a classical observational dimension of the signal view (bispectral frequencies in paper^46^). The frequency domain information could generally reflect how much the signal contains different rate activity components. In the signal decomposition process, 50 trials 2 s segments of each EEG signal were selected for superimposed averaging, resulting in a frequency domain resolution of 0.5 Hz. At the frequency domain level, the five features shown in Supplementary Table 4 were calculated in this study. The following standard frequency bands are used in this work: delta band (0.5–3 Hz), theta band (4–7 Hz), alpha band (8–12 Hz), beta band (13–20 Hz), gamma band (21–40 Hz).

### Spatial domain feature

The spatial features calculate the connections and interactions between different brain regions, reflecting the activity patterns of the nervous system. In this part of the study, the EEG was averaged by superimposing 20 trials 2 s segments, and then the information transfer between individual leads was calculated based on the directed transfer function (DTF) to form a directed graph (calculated with eConnectome toolbox^47^). Subsequent calculations were based on the BCT toolbox^48^ to extract spatial features in the EEG, with the feature parameters shown in Supplementary Table 5. In phase lag index computation, N denotes the time point, ${\varnothing }_{{rel}}$ denotes the phase difference between the two channel signals at time tn, and sign is a sign function.

### Statistical analysis

In the results section of this study, two distinct statistical testing methods were introduced, namely, the two-samples t-test and the Mann–Whitney U Test. Their differentiation lies in the former being employed for assessing differences in samples conforming to a normal distribution, while the latter is utilized for samples that do not adhere to a normal distribution. In this research, an initial Kolmogorov–Smirnov test (K-S test) was conducted on the data distributions to determine which testing method to employ. Specifically, the results of the test indicated that the distributions of the EEG features across different groups followed a normal distribution. As such, the paired-samples t-test was employed for these datasets, where a *p*-value less than 0.1 was considered indicative of a significant difference, and a *p*-value less than 0.05 was denoted with an asterisk (*). Conversely, the Kolmogorov–Smirnov test results indicated that the distribution of scores on the cognitive assessment scale did not adhere to a normal distribution. Consequently, the Mann–Whitney U Test was applied to analyze this particular subset of data. Similar to the paired-samples t-test, the results of the Wilcoxon signed-rank test considered a *p*-value less than 0.1 as indicative of a significant difference and a *p*-value less than 0.05 was denoted with an asterisk (*).

### Feature selection and model training

In this study, the EEG features of HC, MCI, and dementia were extracted from three spatial and temporal frequency perspectives, which describe the differential information contained in the EEG from different perspectives. Many features are extracted from the research’s time, space, and frequency domains, and each feature may contain multiple parameters. To avoid excessive spatiotemporal complexity, this study uses the ReliefF^49^ feature selection method, which is a method of assigning different weights to features by considering the distance between like and unlike, and the core idea of ReliefF is that if a feature makes the closest distance of a sample point to like less than the closest distance of unlike, then the feature is beneficial for classification and the weight of the feature is increased. This study uses the ReliefF method. This study uses the ReliefF method to rank the features in descending order of weight and selects the top N features that maximize the correct classification rate as the final training result of the model.

Different information has different abilities to differentiate between different groups of people at the feature level, so a specific approach is needed to combine and train the valid information to build a cognitive-level assessment model. In terms of feature combination, SVM was first used to construct models for Fractal characteristics, Complexity measures, Microstate features, Spectral characteristics, Bispectral features, Network Properties, and Phase synchronization, and eight weak classifiers was constructed. This study combined the eight weak classifiers into one strong classifier for HC, MCI, and dementia classification using the AdaBoost idea. The SVM method was also used to predict the three categories’ EEG and cognitive scale scores, and the results were combined using Adaboost.

AdaBoost procedure (Classification and regression):

Step 1: Each sample is given an initial weight *w* = 1/*N* (a total of *N* samples), and all weak classifiers are used for classification.

Step 2: Select the classifier with the smallest error rate (the sum of the weights of all misclassified sample points is the error rate e) so that its weight is 0.5*log((2−e)/e).

step3: Update the sample weights, the correct sample weight is *w*/2*(1−e), the wrong sample weight is *w*/2*e.

Step 4: Repeat Step 2 until all classifiers are assigned weights or several iterations are reached.

According to previous studies, healthy individuals, mild cognitive impairment, and dementia are a continuum of development and are accompanied by a gradual decline in cognitive ability^50,51^. This means that these three groups represent the full range of the cognitive level space, and if the interval 0–100 is used as the cognitive level score, the healthy group would have the highest score, the mild cognitive impairment group would have the medium score, and the dementia group would have the lowest score. Based on this criterion, the study divided the 100 equivalents into three ranges. That is, 66.6–100 for the normal group, 33.3–66.6 for the mild cognitive impairment group, and 0–33.3 for the dementia group.

In this study end of the AdaBoost algorithm, all classifiers were given weights, and the final cognitive assessment scores were obtained by summing the classification and cognitive regression results. The final cognitive scores are shown in Formula 2, with the classification results being a default score of 66.6 for HC, 33.3 for MCI, and 0 for dementia.

2 $$\begin{array}{l}{\rm{ognitive}}\; {\rm{score}}={\rm{classification}}\; {\rm{results}}+\,0.5\\\qquad\qquad\qquad\quad* ({\rm{MoCAB}}+{\rm{MMSE}})/30* 33.3\end{array}$$

The formula implies that the scale scores obtained by EEG fitting are first mapped to 0–33 and then mapped to 0–100 according to subjects’ clinical labels (HC, MCI, dementia). The final cognitive scores are shown in Eq. 1, with a default score of 66.6 for HC, 33.3 for MCI, and 0 for dementia. In the formula, 30 represents a score of 30 on the cognitive scale. 0.5 represents the mean value of the MoCAB and MMSE scores.

### Model validation

To validate the predictive performance of the model, this study introduced two validation components. Firstly, the research conducted cross-validation on an internal dataset, wherein 10 healthy subject samples, 10 mild cognitive impairment patient samples, and 10 dementia patient samples, totaling 30 samples, were reserved as the test set in each round, without participating in the training. 504 EEG cases were included in the study, of which 474 cases were used as training samples and 30 cases were set aside as test samples. The following principles were followed when reserving the test sample: 1. random sampling. 2. 10 cases from HC, 10 from MCI, and 10 from dementia. 3. ensure no sample was taken from the same subject twice. Otherwise, re-sample. After a total of 15 rounds of cross-validation, the average error was calculated as the final prediction result. The second validation component involved testing on an external dataset, consisting of 69 samples, all of which were used as test samples.

### Reporting summary

Further information on research design is available in the Nature Research Reporting Summary linked to this article.

### Supplementary information

Supplementary Information files

Reporting Summary

## Data Availability

The datasets used or analyzed during the current study are available from the corresponding author upon reasonable request.

## Code availability

The analysis code is available from public toolbox described in the article, and the code of integrated learning section is uploaded at https://github.com/Sun199510/adaboost_matlab.

## References

[1] Petersen RC (1999). Mild cognitive impairment - clinical characterization and outcome. Arch. Neurol..

[2] Petersen RC (2004). Mild cognitive impairment as a diagnostic entity. J. Int. Med..

[3] Charles ST, Carstensen LL (2010). Social and emotional aging. Ann. Rev. Psychol..

[4] Gauthier S (2006). Mild cognitive impairment. Lancet.

[5] Rao SM, Leo GJ, Bernardin L, Unverzagt F (1991). Cognitive dysfunction in multiple-sclerosis. 1. Frequency, patterns, and prediction. Neurology.

[6] West RL (1996). An application of prefrontal cortex function theory to cognitive aging. Psychol. Bull..

[7] Crum RM, Anthony JC, Bassett SS, Folstein MF (1993). Population-based norms for the mini-mental-state-examination by age and educational-level. Jama.

[8] Thapa, N. et al. The effect of a virtual reality-based intervention program on cognition in older adults with mild cognitive impairment: a randomized control trial. *J. Clin. Med.*10.3390/jcm9051283 (2020). 10.3390/jcm9051283 PMC7288029 32365533

[9] Albert MS (2011). The diagnosis of mild cognitive impairment due to Alzheimer’s disease: recommendations from the National Institute on Aging-Alzheimer’s Association workgroups on diagnostic guidelines for Alzheimer’s disease. Alzheimers Dementia.

[10] Sheehan B (2012). Assessment scales in dementia. Ther. Adv. Neurol. Disord..

[11] Tison F (2000). Contribution and limitations of evaluation scales in Parkinson’s disease. Rev. Neurol..

[12] Ismail Z, Rajji TK, Shulman KI (2010). Brief cognitive screening instruments: an update. Int. J. Geriatr. Psychiatry.

[13] Lee KH, Kim H-K (2008). Limitations of mini mental state examination in assessing cognitive functions of Korean older adults. Korean J. Psychol. General.

[14] Morrell L, Camic PM, Genis M (2019). Factors associated with informant-reported cognitive decline in older adults: a systemised literature review. Dementia.

[15] Jeong JS (2004). EEG dynamics in patients with Alzheimer’s disease. Clin. Neurophysiol..

[16] Brown RE, Basheer R, McKenna JT, Strecker RE, McCarley RW (2012). Control of sleep and wakefulness. Physiol. Rev..

[17] Palop JJ, Mucke L (2009). Epilepsy and cognitive impairments in Alzheimer’s disease. Arch. Neurol..

[18] D’Rozario, A. L. et al. Objective measurement of sleep in mild cognitive impairment: a systematic review and meta-analysis. *Sleep Med. Rev.*10.1016/j.smrv.2020.101308 (2020). 10.1016/j.smrv.2020.101308 32302775

[19] Jafari, Z., Kolb, B. E. & Mohajerani, M. H. Neural oscillations and brain stimulation in Alzheimer’s disease. *Prog. Neurobiol.*10.1016/j.pneurobio.2020.101878 (2020). 10.1016/j.pneurobio.2020.101878 32615147

[20] Greco C (2021). Discriminative power of EEG-based biomarkers in major depressive disorder: a systematic review. IEEE Access.

[21] Parker AF (2022). A systematic review of neuroimaging studies comparing individuals with subjective cognitive decline to healthy controls. J. Alzheimers Dis..

[22] Ieracitano C, Mammone N, Hussain A, Morabito FC (2020). A novel multi-modal machine learning based approach for automatic classification of EEG recordings in dementia. Neural Netw..

[23] Townley, R. A. et al. Progressive dysexecutive syndrome due to Alzheimer’s disease: a description of 55 cases and comparison to other phenotypes. *Brain Commun.*10.1093/braincomms/fcaa068 (2020). 10.1093/braincomms/fcaa068 PMC7325839 32671341

[24] Aarsland, D. et al. Parkinson disease-associated cognitive impairment. *Nat. Rev. Dis. Primers*10.1038/s41572-021-00280-3 (2021). 10.1038/s41572-021-00280-3 34210995

[25] Babiloni C (2021). Measures of resting state EEG rhythms for clinical trials in Alzheimer’s disease: recommendations of an expert panel. Alzheimers Dement..

[26] Tzimourta, K. D. et al. Machine learning algorithms and statistical approaches for Alzheimer’s disease analysis based on resting-state EEG recordings: a systematic review. *Int. J. Neural Syst.*10.1142/s0129065721300023 (2021). 10.1142/S0129065721300023 33588710

[27] Schapire RE (1990). The strength of weak learnability. Mach. Learn..

[28] Khanna A, Pascual-Leone A, Michel CM, Farzan F (2015). Microstates in resting-state EEG: current status and future directions. Neurosci. Biobehav. Rev..

[29] Elmore S (2007). Apoptosis: a review of programmed cell death. Toxicol. Pathol..

[30] Cheng HY, Weng CC, Chen YY (2012). Vehicle detection in aerial surveillance using dynamic bayesian networks. IEEE Trans. Image Process..

[31] Sun JN, He J, Gao XR (2021). Neurofeedback training of the control network improves children’s performance with an SSVEP-based BCI. Neuroscience.

[32] Cabeza R, Anderson ND, Locantore JK, McIntosh AR (2002). Aging gracefully: compensatory brain activity in high-performing older adults. Neuroimage.

[33] Wolz, R. et al. Multi-method analysis of MRI images in early diagnostics of Alzheimer’s disease. *PLoS ONE*10.1371/journal.pone.0025446 (2011). 10.1371/journal.pone.0025446 PMC3192759 22022397

[34] Petersen, S. E. & Posner, M. I. In *Annual Review of Neuroscience* Vol. 35 (ed. Hyman, S. E.) 73–89 (2012). 10.1146/annurev-neuro-062111-150525 PMC3413263 22524787

[35] Patterson, R. A. et al. Neurophysiological and other features of working memory in older adults at risk for dementia. *Cogn. Neurodynamics*10.1007/s11571-023-09938-y (2023). 10.1007/s11571-023-09938-y PMC11143125 38826646

[36] Buckner RL (2009). Cortical hubs revealed by intrinsic functional connectivity: mapping, assessment of stability, and relation to Alzheimer’s disease. J. Neurosci..

[37] Rossini PM (2020). Early diagnosis of Alzheimer’s disease: the role of biomarkers including advanced EEG signal analysis. Report from the IFCN-sponsored panel of experts. Clin. Neurophysiol..

[38] Alyasseri, Z. A. A. et al. Multi-objective flower pollination algorithm: a new technique for EEG signal denoising. *Neural Comput. Appl.*10.1007/s00521-021-06757-2 (2022).

[39] Sun YK (2022). Minimally invasive local-skull electrophysiological modification with piezoelectric drill. IEEE Trans. Neural Syst. Rehabilitation Eng..

[40] Xiao, S. S., Li, Y. J., Liu, M. & Li, Y. X. Electrophysiological studies of cognitive reappraisal success and failure in aMCI. *Brain Sci.*10.3390/brainsci11070855 (2021). 10.3390/brainsci11070855 PMC8301780 34198957

[41] Cejnek M, Vysata O, Valis M, Bukovsky I (2021). Novelty detection-based approach for Alzheimer’s disease and mild cognitive impairment diagnosis from EEG. Med. Biol. Eng. Comput..

[42] Nasreddine ZS (2005). The montreal cognitive assessment, MoCA: a brief screening tool for mild cognitive impairment. J. Am. Geriatr. Soc..

[43] Tombaugh TN, McIntyre NJ (1992). The mini-mental-state-examination—a comprehensive review. J. Am. Geriatr. Soc..

[44] Lempel A, Ziv J (1976). Complexity of finite sequences. IEEE Trans. Info. Theory.

[45] Fulcher B, Jones NS (2017). hctsa: a computational framework for automated time-series phenotyping using massive feature extraction. Cell Syst..

[46] Glass PS (1997). Bispectral analysis measures sedation and memory effects of propofol, midazolam, isoflurane, and alfentanil in healthy volunteers. Anesthesiology.

[47] He B (2011). eConnectome: A MATLAB toolbox for mapping and imaging of brain functional connectivity. J. Neurosci. Methods.

[48] Rubinov M, Sporns O (2010). Complex network measures of brain connectivity: uses and interpretations. Neuroimage.

[49] Robnik-Sikonja M, Kononenko I (2003). Theoretical and empirical analysis of ReliefF and RReliefF. Mach. Learn..

[50] Langa KM, Levine DA (2014). The diagnosis and management of mild cognitive impairment a clinical review. Jama.

[51] Morris JC (2001). Mild cognitive impairment represents early-stage Alzheimer disease. Arch. Neurol..

