# Supplementary material for: An ensemble learning model for continuous cognition assessment based on resting-state EEG
Source: NPJ Aging. 2024 Jan 2;10(1):1. doi: 10.1038/s41514-023-00129-x (PMC10762083; doi:10.1038/s41514-023-00129-x)
Supplement: Supplementary file 1 — Supplementary Information files [file 41514_2023_129_MOESM1_ESM.pdf]

Supplementary Information

An Ensemble Learning Model for Continuous Cognition Assessment Based on Resting-State EEG

Sun Jingnan[1], Sun Yike[1], Shen Anruo[1, 2], Li Yunxia[5], Gao Xiaorong[1, *], Lu Bai[3, 4, *]

[1] Department of Biomedical Engineering, Tsinghua University, China, 100084
[2] Department of Biomedical Engineering, Johns Hopkins University, Baltimore, MD, USA, 21218
[3] School of Pharmaceutical Sciences, IDG/McGovern Institute for Brain Research, Tsinghua-Peking Joint Center for Life Sciences, Tsinghua University, Beijing, China, 100084
[4] Advanced Innovation Center for Human Brain Protection, Capital Medical University, Beijing, China,100070
[5] Department of Neurology, Tongji Hospital, School of Medicine, Tongji University, Shanghai, China, 200092

*** Correspondence:**

Dr. Xiaorong Gao, Department of Biomedical Engineering, Tsinghua University, Beijing 100084, China, E-mail: gxr-dea@tsinghua.edu.cn
Dr. Bai Lu, School of Pharmaceutical Sciences, Room B303, Tsinghua University, Beijing 100084, China, Tel: +86 62785101, E-mail: bai_lu@tsinghua.edu.cn

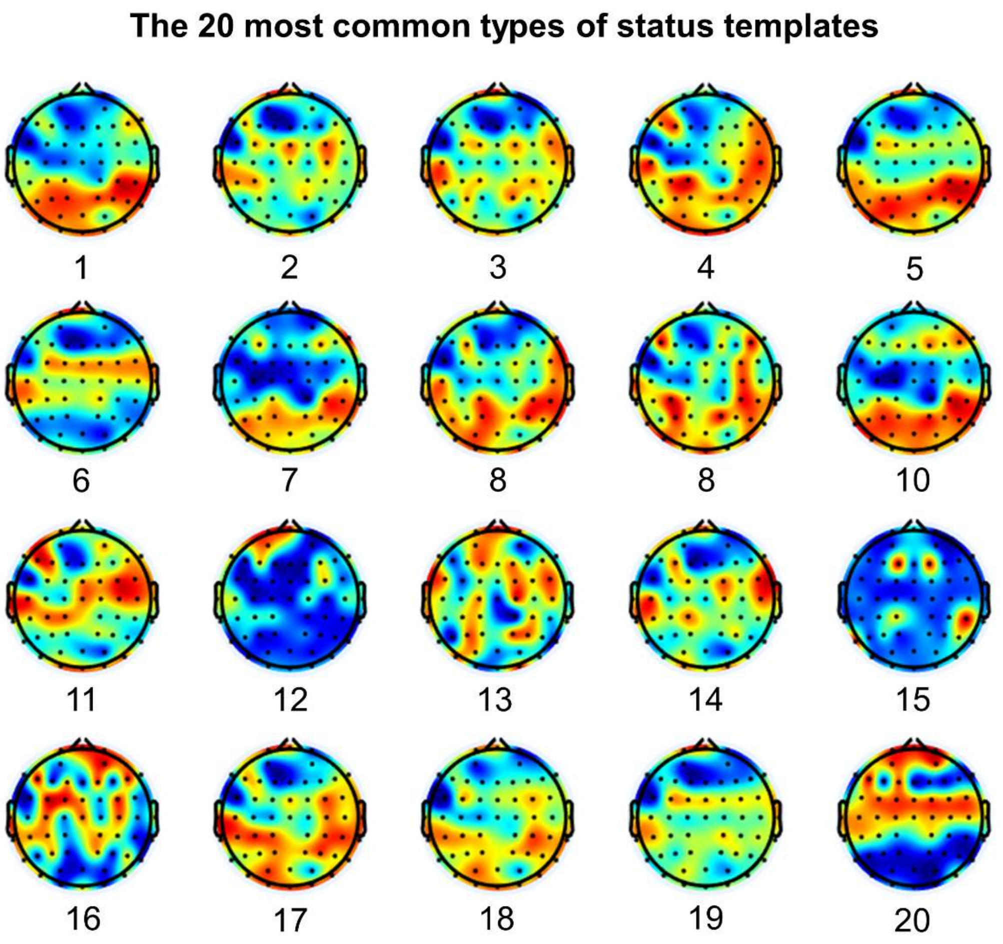

Supplementary Figure 1. Common microstate divisions. 20 common microstates were classified according to patterns of neural activity in three categories of people. Too few microstates would result in poor spatial resolution, while too many would lack representation. The results show that State 4 is very similar to the Central Executive Network (CEN), while State 5 is closer to the Sensorimotor System Network (SSN). State 15 has some similarities to the Default Mode Network (DMN). EEG microstates depict the spatial pattern of cerebral activity at a fine level of electrical activity over time, and their relevance against the theoretical network basis of scalar function MRI might be evaluated. Of note is the absence of states 12 and 15 in patients with dementia.

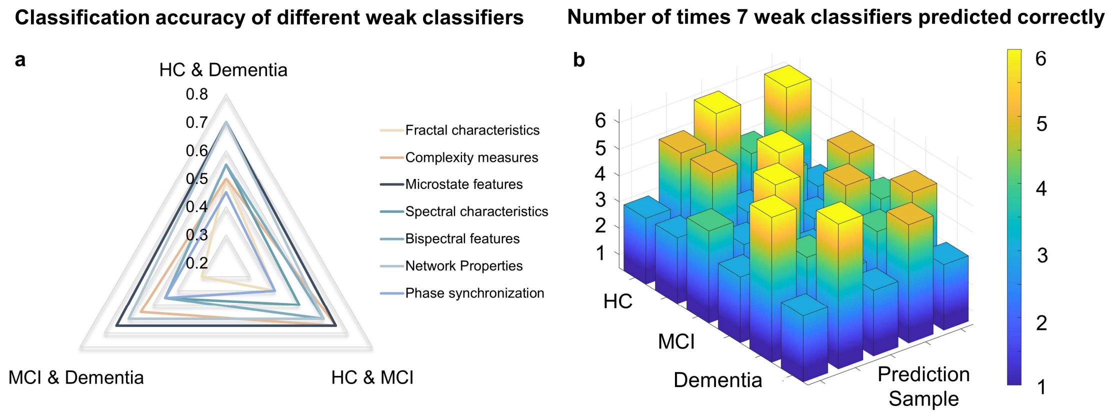

Supplementary Figure 2. Performance of weak classifiers. The results showed that the seven weak classifiers did not have the same ability to discriminate between the three categories, and the weak classifiers performed relatively well in discriminating between HC and dementia. **a** In the distinction between HC and MCI, the micro-state features performed the best, reaching an accuracy of 0.66. Moreover, in the distinction between MCI and dementia, the network attribute performed the best, obtaining an accuracy of 0.68 for classification. **b** The figure on the right shows the performance of the 30 test samples under seven wake classifiers. Where the histogram indicates the number of times it was predicted correctly, for each sample point, it could be an integer between 0 and 7. The shades of color indicate the magnitude of the value.

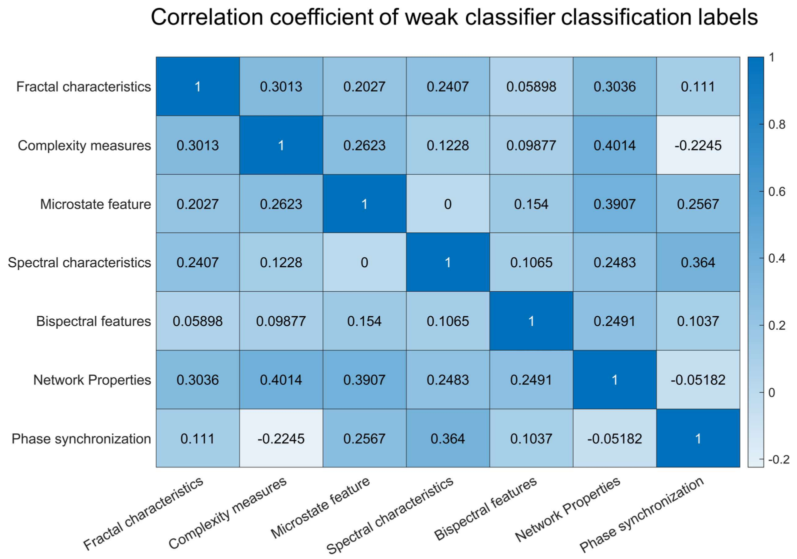

Supplementary Figure 3. Correlation of weak classifier results. The results of the correlation analysis of the classification label results for each weak classifier pointed out that no two weak classifiers' results presented a high correlation. The coefficient matrix shows that the highest correlation coefficient between complexity features and network features is 0.40, which cannot be considered a strong correlation. This result indicates that each classifier performs differently and has no redundant information.

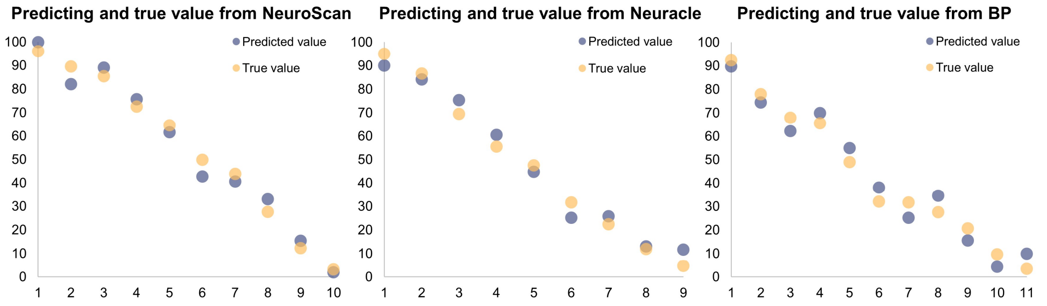

Supplementary Figure 4. Among the 30 test samples set aside, 10 were from NeuroScan, 9 from Neuracle, and 11 from amplifier BP. The results show no difference between the distribution of the test samples and the predicted samples.

Supplementary Table 1. Predictive distribution test for different amplifiers

Amplifiers	Mean absolute error	True Value Distribution Inspection	Predicted Value Distribution Inspection
NeuroScan	4.13	NeuroScan & Neuracle p=0.32	NeuroScan & Neuracle p=0.37
Nuracle	4.33	Neuracle & BP p=0.40	Neuracle & BP p=0.31
BP	5.23	NeuroScan & BP p=0.21	NeuroScan & BP p=0.20

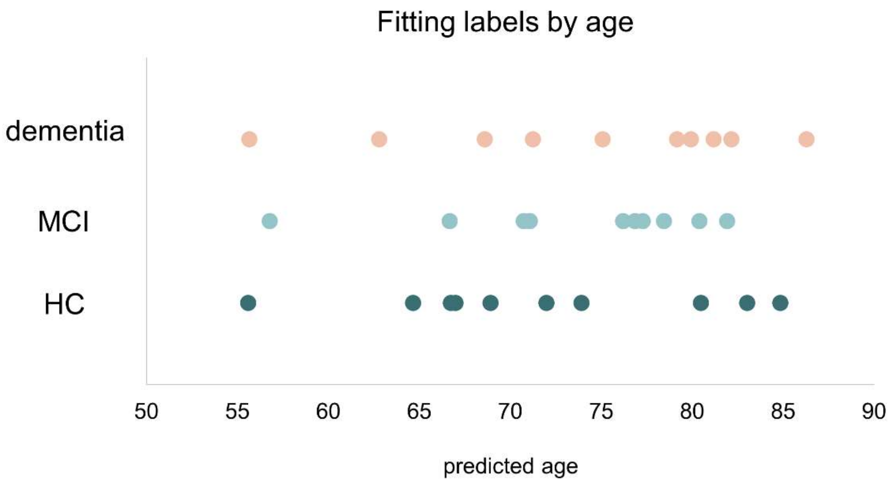

Supplementary Figure 5. Result of fitting labels by age. The actual clinical labels were drawn using the same method (AdaBoost) for age fitting. The results point out that there is a certain age factor in the prediction model proposed in this paper, but the cognitive level does not depend entirely on age.

Supplementary Table 2. Fractal features

Feature	Description	Calculation
Peak value (pk)	Difference between the maximum and minimum values	max(X)-min(X)
Rectification average value (av)	Average activity intensity	$\overline{\lvert X \rvert}$
Effective value (rm)	Description of power	$\sqrt{\overline{X^2}}$
kurtosis	The extent that the signal deviates from a normal distribution	$\frac{\frac{1}{n}\sum_{i=1}^{n}(X_i-\bar{X})^4}{(\frac{1}{n}\sum_{i=1}^{n}(X_i-\bar{X})^2)^2}$
skewness	Signal symmetry	$(\frac{\frac{1}{n}\sum_{i=1}^{n}(X_i-\bar{X})^3}{\frac{1}{n}\sum_{i=1}^{n}(X_i-\bar{X})^2})^{\frac{3}{2}}$
margin	Maximum deviation	$\frac{pk}{\overline{\sqrt{\lvert X \rvert}}^2}$
form factor	Shock characteristics of the signal	$\frac{rm}{av}$
Impulse Factor	Shock characteristics of the signal	$\frac{pk}{av}$
crest factor	extremes of peaks in the signal	$\frac{pk}{rm}$

Supplementary Table 3. Microstate sequence features

Feature	Description	Calculation
occurrences	Number of occurrences of each state	$\sum S_i$
Status transformation	Number of interconversions between any two types of states	$\sum_{i=1}^{20}\sum_{j=1}^{20} S_i \rightarrow S_j$
Conversion distance	Distance between two mutually transitional states	$\sqrt{\sum_{1}^{n}(S_{in} - S_{jn})^2}$
Distribution entropy	Distribution properties of sequences	hctsa toolbox[45]
Approximate entropy	Degree of self-similarity of sequences	hctsa toolbox[45]
auto-mutual information change	Changes in the auto-mutual information with the addition of noise.	hctsa toolbox[45]
generalized linear self-correlation	The generalized linear self-correlation function of a time series.	hctsa toolbox[45]
AR model	Fits an AR model	hctsa toolbox[45]
GARCH model	Fits an GARCH model	hctsa toolbox[45]
KPSS stationarity	The KPSS stationarity test	hctsa toolbox[45]
PP stationarity	Phillips-Peron unit root test	hctsa toolbox[45]
Lyapunov index	Dispersion rate of chaotic systems	hctsa toolbox[45]
Poincare section	Parametric description of non-linear systems	hctsa toolbox[45]
binary statistics	Statistics on a binary symbolization of the time series.	hctsa toolbox[45]

Supplementary Table 4. Frequency features

Feature	Description	Calculation
Fast Fourier Transform	Frequency domain power distribution	$fft(X)$
Center of gravity	The point at which the sum of the	0.5*$\sum fft(X)$
RMS frequency	root mean square of the spectrum	$\sqrt{X * fft(X)^2}$
variance frequency	frequency of variance	$std(fft(X))$
Bispectral frequencies	Signal symmetry	Method in paper[46]

Supplementary Table 5. Spatial domain features

Feature	Description	Calculation
directed transfer function	Directed information flow of different signal	eConnectome toolbox [47]
Phase lag index	The degree of phase synchronization of the two signals	$\lvert \frac{1}{N}\sum_{n=1}^{N} sign(\Delta\emptyset_{rel}(t_n)) \rvert$
Node degree	Number of neighbors connected to the node	BCT toolbox [48]
Node strength	Weighted node degree considering connection strength	BCT toolbox [48]
Clustering coefficient	Network local information processing efficiency	BCT toolbox [48]
Average path	The average value of the shortest path to pass information between any two	BCT toolbox [48]
Small World Properties	Distributed information processing capability within the network	BCT toolbox [48]
Rich Club Properties	Network core critical node interaction capability	BCT toolbox [48]
